# Sex Differences in Long-Term Potentiation at Temporoammonic-CA1 Synapses: Potential Implications for Memory Consolidation

**DOI:** 10.1371/journal.pone.0165891

**Published:** 2016-11-02

**Authors:** Xiaoqiang Qi, Ke Zhang, Ting Xu, Vitor Nagai Yamaki, Zhisheng Wei, Mingfa Huang, Gregory M. Rose, Xiang Cai

**Affiliations:** 1 Department of Physiology, Southern Illinois University School of Medicine, Carbondale, IL, United States of America; 2 The Institute of Neuroscience, Key Laboratory of Neurogenetics and Channelopathies of Guangdong Province and the Ministry of Education of China, The Second Affiliated Hospital of Guangzhou Medical University, Guangzhou, P.R. China; 3 Department of Anatomy, Southern Illinois University School of Medicine, Carbondale, IL, United States of America; 4 Neuroscience Research Center, Southern Illinois University School of Medicine, Carbondale, IL, United States of America; Bilkent University, TURKEY

## Abstract

Sex differences in spatial memory have long been observed in humans, non-human primates and rodents, but the underlying cellular and molecular mechanisms responsible for these differences remain obscure. In the present study we found that adolescent male rats outperformed female rats in 7 d and 28 d retention probes, but not in learning trials and immediate probes, in the Morris water maze task. Male rats also had larger long-term potentiation (LTP) at hippocampal temproammonic-CA1 (TA-CA1) synapses, which have been implicated to play a key role in place field and memory consolidation, when protocols designed to elicit late-stage LTP (LLTP) were used. Interestingly, the ratio of evoked AMPA/NMDA currents was found to be smaller at TA-CA1 synapses in male rats compared to female rats. Protein biotinylation experiments showed that male rats expressed more surface GluN1 receptors in hippocampal CA1 stratum lacunosum-moleculare (SLM) than female rats, although GluA1 expression was also slightly higher in male rats. Taken together, our results suggest that differences in the expression of AMPA and NMDA receptors may affect LTP expression at TA-CA1 synapses in adolescent male and female rats, and thus possibly contribute to the observed sex difference in spatial memory.

## Introduction

Spatial navigation in familiar or new environments is an essential ability for many species in their daily life [1]. In humans, as early as infancy, children have the ability to localize spatial information based on the relationship between an event and environmental features [2,3]. Studies show that a significant improvement in the ability to process spatial and contextual information occurs in adolescence, the consequence of rapid structural and functional changes in the brain from childhood into adolescence [3]. Sex differences in performing spatial tasks have long been observed in humans, non-human primates and rodents [4–9], but the underlying mechanisms are not fully understood. Several studies have suggested that the brain regions that are associated with spatial cognitive processes could be different, or functioning differentially, in each sex [10–14]. However, the key neural circuits and biochemical signaling pathways responsible for these discrepancies are still largely unidentified.

There is considerable evidence suggesting that the hippocampus is involved in episodic and spatial memory [15–19]. Sexual dimorphism in hippocampus-dependent behaviors suggests that there could be differences in the anatomical organization of hippocampal circuitry in males and females. However, a several studies have failed to reveal significant differences in hippocampal volume between men and women after correcting for total intracranial volume [20–23], although some have reported a larger hippocampus in women than in men [24,25]. In addition, when the anterior and posterior hippocampal regions were considered separately, one study reported that women exhibited a larger posterior hippocampus than men after normalizing to brain size [22].

On the other hand, in view of the proposed role of synaptic plasticity processes in learning and memory, another possibility is that sex differences in hippocampus-dependent tasks could result from dissimilarities in hippocampal LTP induction or expression between males and females. While this hypothesis has not been extensively explored, studies to date have observed sex differences in the magnitude of LTP at entorhinal inputs to the dentate gyrus, the mossy fiber connection to area CA3, and Schaffer collateral inputs to area CA1 [26–31].

The pyramidal neurons in hippocampal CA1 receive two types of input from the entorhinal cortex. Neurons in layer II of the entorhinal cortex project indirectly to CA1 via the dentate gyrus (DG) and CA3 region through the perforant path, while neurons in entorhinal layer III primarily terminate on the distal dendrites of pyramidal neurons in CA1 and the subiculum. This monosynaptic pathway from the entorhinal cortex to CA1 is called the temporoammonic pathway (TA). Studies show that lesioning the TA inputs from the entorhinal cortex to CA1 region did not affect the formation of hippocampal memories, but prevented them from becoming consolidated in cortical networks[32,33]. When inputs from area CA3 were removed to isolate the TA-CA1 circuit, place fields of CA1 neurons were preserved, and rats with an isolated CA1 area performed normally in spatial recognition tasks, but were impaired in spatial navigation[34]. These observations suggest that the direct entorhinal-hippocampal connections play a unique and pivotal role in spatial learning and memory. The potential involvement of the TA pathway in sex differences in spatial navigation has not been previously examined. In the present study, we found that adolescent male rats outperformed female rats in retention probes during Morris water maze (MWM) task, a test of spatial memory. Male rats also showed larger magnitude LTP of the TA pathway, consistent with lower AMPA/NMDA receptor ratios at TA-CA1 synapses in males versus females.

## Methods

### Animals

Three-week old male and female Sprague-Dawley pups were purchased as litters with dams from Harlan Laboratories (Indianapolis, IN, USA). The number of litters was decided by the number of animals needed for experiments and litter size; usually 2–4 litters were ordered at one time. Pups were weaned upon arrival and then housed in clear Plexiglas cages (46 x 20 x 23 cm, 3 rats/cage, male and female rats housed separately) with wood chip bedding and maintained on a 12-hour light/dark cycle. The rats were given food and water ad libitum. Experiments were performed between P42 and P77. All experimental procedures involving animals in this study were approved by the Animal Care Committee of Southern Illinois University, which operates according to policies consistent with NIH guidelines for the care and use of laboratory animals. With the exception of the results shown in the effect of estrogen on the magnitude of theta burst LTP, male and female littermates were compared in all experiments.

### Morris water maze test

The water maze consisted of a circular pool (1.6 m diameter; 0.6 m height) filled with tepid water (22 ± 1°C) made opaque by the addition of light gray non-toxic paint. A circular Plexiglas escape platform (14.5 cm in diameter) was located in the center of one of the quadrants (northwest quadrant in this study) of the pool. A blue curtain was located on one side of the pool to separate it from a computer-based video tracking system used for data acquisition (AnyMaze, San Diego Instruments, San Diego, CA). Large black shapes were painted on other walls of the water maze room to serve as additional visual cues. The animals were given 15 trials (acquisition trials) over 5 consecutive days with the platform submerged 2 cm below the surface of the water (three trials per day; 120 s maximum trial duration; 20–30 min inter-trial interval). For each trial, rats were placed at one of three starting locations in pseudorandom order (northeast, southwest, southeast), and were allowed to swim until they located the platform. Rats failing to find the platform within 120 s were hand guided to it. After reaching the platform the animal remained on it for 20 s, after which it was removed from the pool, dried with a towel and placed back into its home cage. Latency and swim distance to find the platform were recorded for learning trials. To assess the memory, probe sessions were performed immediately, 24 h, 7 d and 28 d after the last acquisition trial. For probe trials the platform was removed and rats were placed in the water at the starting location opposite the goal quadrant and allowed to swim for 60 s. The time spent swimming in the goal quadrant, latency to the first cross over the location where the platform had been, and the mean distance (sampled 10 times/s) from the platform location were recorded.

### Acute slice electrophysiology

These methods have been described in detail in our previous publications [35,36]. Rats were killed by decapitation after sedation with Euthasol solution (0.5 ml/kg, intraperitoneally;this solution contains 390 mg/ml sodium pentobarbital and 50mg/ml phenytoin sodium; Henry Schein, Melville, NY). Hippocampi were isolated and sectioned into 400-μm-thick slices in ice-cold artificial cerebrospinal fluid (ACSF) (in mM: 124 NaCl, 3 KCl, 1.25 NaH_2_PO4, 1.5 MgCl_2_, 2.5 CaCl_2_, 26 NaHCO3, and 10 glucose, bubbled with 95% O2/5% CO2) using a Leica VT 1200S vibratome (Leica Microsystems Inc., Bannockburn, IL, USA). The slices were maintained at room temperature for over 1 h in an interface holding chamber in a humidified atmosphere saturated with 95% O2/5% CO2. The slices were then transferred to a submersion-type recording chamber and perfused at room temperature with ACSF (flow rate 1–2 ml/min). Concentric bipolar tungsten electrodes were placed either in SLM to stimulate TA afferents or in SR to stimulate SC afferents. Extracellular recording pipettes were filled with ACSF (tip resistances 2–5 MΩ) and placed in the same layer (e.g., in SLM to record TA-CA1 responses), 100–150 μm from the stimulating electrodes. Stimuli (100 μs duration) were delivered at 0.05 Hz. The stimulus intensity was set at 150% of the threshold intensity, resulting in a fEPSP of 0.1–0.4 mV. All compounds were applied by perfusion. fEPSPs were recorded using an Axoclamp 2B amplifier (Molecular Devices, Sunnyvale, CA, USA); responses were low-pass filtered at 10 kHz and amplified 100× prior to digitization. Three LTP induction protocols were employed: a single train of 100 pulses at 100 Hz was used to generate early LTP (ELTP); 4 x HFS, consisting of 4 trains of 100 pulses given at 100 Hz, with a 5 min inter-train interval, and TBS, consisting of 4 trains of 10 bursts at 5 Hz, with each burst contained 4 stimuli at 100 Hz, with a 10 s inter-train interval, were used to generate late LTP (LLTP). The magnitude of LTP was assessed by measuring average of peak amplitudes of fEPSPs (% of last 10 min of baseline) at 51–60 min after LTP stimulation was given.

Whole-cell voltage clamp recordings were obtained with patch pipettes filled with (in mM): CsCH_3_SO_3_ 135, HEPES 10, NaCl 10, MgCl_2_ 1, K_4_BAPTA 0.1, Mg^2+^-ATP 2, and phosphocreatine 10, and QX-314 adjusted to pH 7.3 with CsOH. In whole-cell recording experiments, picrotoxin (100 μM) and CGP52432 (4 μM) were included in the ACSF to block GABA_A_ and GABA_B_ receptors respectively. Area CA3 and the DG were removed from the slice to prevent spontaneous epileptiform discharges. Hippocampal CA1 pyramidal cells were illuminated with near-infrared (IR) light and visualized with a 40x water-immersion lens; images were detected with an IR-sensitive CCD camera. Electrode resistances in the bath were 3–6 MΩ, and series resistances of <40 MΩ were accepted. Data were collected using a Multiclamp 700B or an Axopatch 200B amplifier (Molecular Devices), low-pass filtered at 2k Hz and digitized at 5k Hz using a Digidata 1440A A/D converter and Clampex 10.3 software (Molecular Devices). For calculation of the AMPA/NMDA ratio, the peak amplitude of AMPA receptor-mediated current was measured when the cell membrane potential was held at -70 mV, while NMDA receptor-mediated current was recorded when the cell membrane potential was held at +60 mV and was measured 150 ms after a stimulus.

### Membrane surface protein biotinylation

Hippocampal slices, prepared as described above, were maintained at room temperature for at least 1 h. Area CA1 SLM wedges were dissected from backlit hippocampal slices with the aid of a dissecting microscope using the clearly identifiable landmarks of the hippocampal fissure and the proximal border of the TA fibers in the distal CA1 dendritic region. The SLM samples were transferred to ice-cold ACSF (bubbled with 95% O_2_/5% CO_2_) containing 1 mg/ml sulfosuccinimidyl-2-(biotinamido) ethyldithio-propionate (sulfo-NHS-SS-biotin; Fisher Scientific, Pittsburgh, PA, USA) for 1 h to biotinylate surface proteins. Excess biotin was then removed by washing the slices 3 times with ice-cold ACSF to eliminate free sulfo-NHS-SS-biotin. Twelve CA1 SLM samples were pooled and homogenized in 600 μl of modified RIPA buffer (Fisher Scientific) containing 150 mM NaCl, 20 mM Hepes, 1% Triton X-100, 0.5% SDS and 2 mM EDTA (pH 7.4), supplemented with a cocktail of protease and phosphatase inhibitors. The tissue was homogenized for 30 s and placed on ice under agitation for 1 h. Homogenates (total fractions) were collected, and cellular debris was removed by centrifugation for 10 min at 13,000 rpm at 4°C, after which the supernatants were collected. The total protein concentration in the supernatants was analyzed by the bicinchoninic acid assay (BCA assay) and modified RIPA buffer was added as necessary to insure that the protein concentrations were equivalent (3 μg/μl) in all samples. A small fraction (20 μg) of the supernatants was removed for total protein measurement by Western blotting. For surface protein detection, 1.25 mg protein samples were incubated overnight at 4°C with prewashed Neutravidin Agarose beads (50 μl, Fisher Scientific) to capture biotinylated proteins. Beads were recovered by a brief centrifugation and the supernatants were discarded. The sedimented beads were washed 3 times in phosphate buffered saline (PBS) and biotinylated proteins were finally eluted with loading buffer (boiled at 100°C for 5 min). Biotinylated fractions were collected and levels of surface proteins were estimated by Western blotting. Levels of surface and total GluA1 and GluN1 were expressed as a ratio to actin, which was only detected in total proteins.

### Western blot analysis

Supernatants containing the total proteins (added with loading buffer, boiled at 100°C for 5 min) or biotinylated proteins were loaded into 7.5% Bis-Tris gels (Bio-Rad, Hercules, CA, USA). After running in 1× NuPAGE MOPS SDS buffer (Fisher Scientific), a gel was transferred onto a polyvinylidene difluoride membrane in 1× NuPAGE transfer buffer (in 20% methanol, wt/vol). The membrane was blocked with 5% nonfat dry milk (wt/vol) in buffer containing 1 M Tris-buffered saline and 0.1% Tween-20 (vol/vol) and probed with antibodies to GluA1 (1: 1000, Cell Signaling Technology, Danvers, MA, USA, #13185), and GluN1 (1: 1000, Cell Signaling Technology, #5704) at 4°C overnight. After rinses in TBS-Tween, the membrane was incubated for 1 h at 20–22°C in horseradish peroxidase–conjugated goat antibody to rabbit IgG (1: 3000, Fisher Scientific, #31462). The immunoblot was developed with enhanced chemiluminescence (Fisher Scientific). Levels of expression were computed using ImageJ (NIH). For illustration purposes, blots were cropped and the brightness and contrast were adjusted globally using Photoshop (www.adobe.com).

### Determining the estrous cycle phase

As described by others [37,38], the cycle phase of female rats was obtained through vaginal smears. Vaginal secretions were extracted by mild penetration of the vaginal orifice with a water dropper filled with water and examined under a light microscope. The estrous cycle was determined by the proportion of leukocytes, nucleated epithelial cells and cornified cells. Smears containing predominantly leukocytes (≥ 60%) were classified as diestrus. Smears containing predominantly nucleates epithelial cells (≥60%) and few or no leukocytes (≤ 10%) were classified as proestrus. Smears containing primarily cornified cells (≥ 90%) were classified as estrus.

### Statistical analysis

Data were analyzed using ANOVA, with repeated measures (RMANOVA) where indicated, followed by Bonferroni post hoc analysis, or 2-tailed t-tests as appropriate (GraphPad Prism 5, La Jolla, CA, USA). All data are presented as mean ± s.e.m.. P values of ≤ 0.05 were considered significant.

## Results

**Fig 1:**
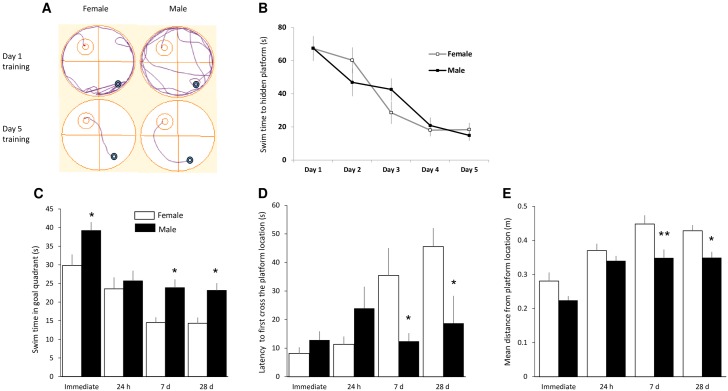
Male rats show better retention of spatial learning in the Morris Water Maze. (A) Representative swim paths show dramatic improvement for both male and female animals from Day 1 (naïve) to Day 5 of training. (B) Mean latencies to find the hidden platform over the 5-day training period. There was no significant difference in the learning curves for male and female animals. (C) Swim times in the goal quadrant during probe trials show that both male and female rats focused their search in the region of the platform’s location during the probe trial given immediately after training. However, males spent more time in the goal quadrant in probe trials given 7d and 28d after training. (D) Time to first cross the platform location during successive probe trials. Times were equivalent for the immediate and 24 h probes, but male rats outperformed females (shorter time to swim to the platform location) 7 d and 28 d after training. (E) Mean distance of the swim path from the platform location, showing shorter mean distance for male rats. n = 6 rats for each sex, *p < 0.05, ** p < 0.01, compared to males.

To evaluate for possible sex differences in spatial learning and memory, adolescent male and female rats were tested in the hidden platform version of the MWM. Both male and female rats quickly improved during 5-consecutive-day training session, as measured by reduced escape latencies [F(4,50) = 26; p < 0.0001; RMANOVA]; there was no difference between the sexes [F(1,50) < 0.01; p > 0.05] (Fig 1A and 1B). To test whether the animals had indeed learned the spatial location of the hidden platform, we removed the platform and ran a probe trial immediately after the final training session. We found that both males and females spent aconsiderable amount of time in the goal quadrant searching for the platform (Fig 1C), had short latencies to cross over the platform’s previous location (Fig 1D), and had small mean swim distances from the platform’s position (Fig 1E), indicating that both groups had learned the platform’s location. To determine whether there was a sex difference in short-term memory or memory consolidation of the learned location of the platform, additional probe tests were performed 24 h, 7 d and 28 d after the final training trial. Compared to the immediate probe, time spent swimming in the goal quadrant decreased, while latency to first cross and mean swim distances from the location of the platform increased, while, in both male and female animals during the delayed probe trials. For time in goal quadrant, RMANOVA revealed significant effects for sex [F(1,40) = 20; p < 0.0001] and time [F(3,40) = 20; p < 0.001], but no interaction [F(3,40) = 1.1; p > 0.05]. Post hoc testing showed that males outperformed females on the immediate probe trial as well as on the probes conducted on days 7 and 28 (p < 0.05 for each). For latency to first cross, RMANOVA revealed a marginal effect for sex [F(1,40) = 3.6; p = 0.06], a significant effect for time [F(3,40) = 4.7; p = 0.006], and a significant interaction [F(3,40) = 5.4; p < 0.005]. Post hoc testing showed that males outperformed females on the probes conducted on days 7 and 28 (p < 0.05 for each). For mean distance from the platform, RMANOVA revealed significant effects for sex [F(1,40) = 22; p < 0.0001] and time [F(3,40) = 22; p < 0.001], but no interaction [F(3,40) = 1.1; p > 0.05]. Post hoc testing showed that males outperformed females on the probes conducted on days 7 (p < 0.01) and 28 (p < 0.05).

Thus, overall performance was not different between male and female groups at the 24 h probe trial. In the 7 d and 28 d probe tests, both male and female animals still actively searched for the platform. However, compared to female rats, the male rats spent more time in the goal quadrant, displayed a shorter latency to first cross the platform location and had smaller mean swim distances from the platform location (Fig 1C–1E), suggesting better memory consolidation of spatial learning.

**Fig 2:**
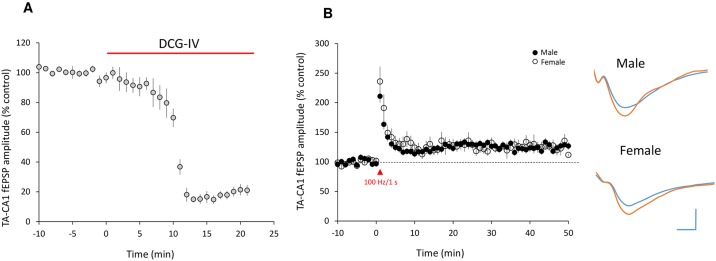
ELTP at TA-CA1 synapses is not different between male and female rats. (A) TA-CA1 fEPSPs were inhibited by DCG-IV (to 19.6 ± 3.5% of baseline, measured 15–20 min after drug application, n = 4 slices from 2 rats. t = 59; p < 0.001). (B) Left—time course of field TA-CA1 EPSPs, showing that ELTP stimulation (1 train, 1s, 100 Hz) induced similar LTP in male and female rats (male, 130.1 ± 11.7% of baseline, measured 40–50 after high frequency stimulation, n = 8 slices from 6 rats; female, 129.5 ± 5.5%, n = 7 slices from 5 rats. t = 0.044; p > 0.05). Right—representative traces before and after LTP. Scale bar: 0.2 mv, 10 ms. Dashed line indicates the baseline response level, which was normalized to 100%.

LTP has been suggested to be a molecular mechanism of learning and memory [39–41]. We therefore compared LTP expression at TA-CA1 synapses in acutely prepared hippocampal slices from male and female animals. Field TA-CA1 EPSPs (fEPSPs) were recorded at hippocampal CA1 SLM synapses by stimulating the TA pathway at 0.05 Hz. Specificity of TA-CA1 responses was verified by demonstrating their near-complete blockade when the selective group II mGluR agonist 2-(2,3-dicarboxycyclopropyl) glycine (DCG-IV; 100 μM) was added to the perfused ACSF (Fig 2A) [42]. After a stable baseline was obtained, TA-CA1 LTP was evaluated.

**Fig 3:**
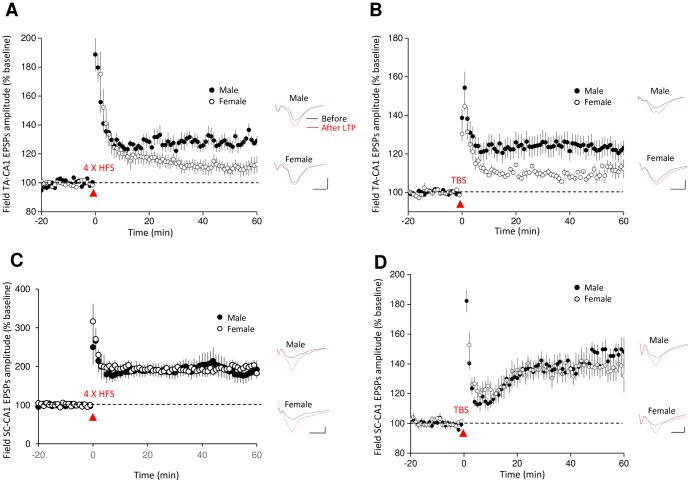
Male rats exhibit larger LLTP at TA-CA1 synapses. (A) Compared with female rats, male rats expressed greater TA-CA1 pathway LTP after high frequency stimulation (HFS, 4 trains of 100 pulses given at 100 Hz, with a 5 min inter-train interval) (130.2 ± 2.1% in males, n = 14 slices from 8 animals; 113.0 ± 3.1% in females, n = 12 slices, 8 animals. t = 4.71, p < 0.0001). (B) Male rats also expressed more TA-CA1 LTP following theta burst stimulation (TBS, 4 trains of 10 bursts at 5 Hz, with each burst consisting of 4 stimuli given at 100 Hz, 10 s inter-train interval) (122.3 ± 3.4% in males, n = 8 slices from 6 animals; 110.0 ± 0.9% in females, n = 9 slices from 7 animals. t = 3.69, p < 0.01). (C) SC-CA1 HFS-induced LTP was not significantly different between male and female rats (194.0 ± 20.2% in males, n = 7 slices from 4 animals; 193.5 ± 24.9% in females, n = 8 slices from 5 animals. t = 0.02, p > 0.05). (D) TBS-induced LTP at SC-CA1 synapses was also not different between male and female rats (143.9 ± 7.4% in males, n = 9 slices from 5 animals; 136.5 ± 8.0% in females, n = 6 slices from 5 animals. t = 0.66, p > 0.05). Representative fEPSPs traces before and after HFS or TBS are shown in each panel; scale bars: 0.2 mv, 10 ms. Dashed lines indicate the baseline response level, which was normalized to 100%. LTP magnitude was quantified as the normalized mean amplitude of fEPSPs measured at 51–60 min.

The ELTP protocol (100 Hz/1 s stimulation) elicited a small, but lasting, increase in TA-CA1 fEPSP amplitude that was not different between slices made from male and female rats (Fig 2B). HFS (4 trains of 100 pulses given at 100 Hz, with a 5 min inter-train interval) also elicited LTP in both male and female rats at TA-CA1 synapses. However, the magnitude of TA-CA1 LTP was significantly greater in slices from male animals compared to female animals (Fig 3A). This result was confirmed using more physiologically patterned stimulation (TBS, see Methods) (Fig 3B). To determine whether this difference was common to other inputs to CA1, we recorded HFS- and TBS-LTP induced by stimulating the Schaffer collateral pathway (SC) in stratum radiatum. We found that there was no difference between males and females in the magnitude of HFS-LTP or TBS-LTP at SC-CA1 synapses (Fig 3C and 3D).

**Fig 4:**
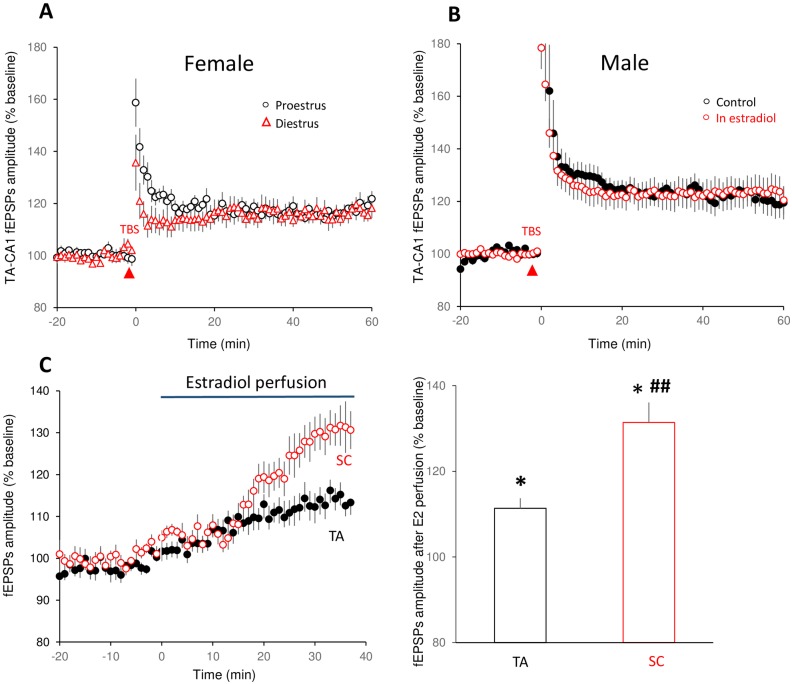
The magnitude of theta burst LTP is not altered by estrogen. (A) There was no difference in TBS-induced TA-CA1 LTP was recorded in slices made from female animals in proestrus versus diestrus (proestrus: 115.6 ± 2.8%, n = 17 slices from 6 animals; diestrus, 114.4 ± 2.8%, n = 16 slices from 9 animals. t = 0.3, p > 0.05). (B) The magnitude of theta burst LTP at TA-CA1 synapses was not affected by estradiol treatment of slices from male rats (estrogen pretreated slices: 122.3 ± 5.4%, n = 31 slices from 24 rats; control slices: 120.9 ± 4.6%, n = 15 slices from 12 rats. t = 0.17, p > 0.05). (C) Time course (left) and data summary (right) illustrate that estradiol (1 nM) application to hippocampal slices from male rats resulted in a large increase in SC-CA1 fEPSPs (131.6 ± 4.7%, n = 10 slices from 6 animals) but less of an increase of TA-CA1 fEPSPs (111.4 ± 2.3%, n = 13 slices from 10 animals). Incubation period with estradiol (approximately 30 min) is indicated by the shaded area. *p < 0.05, compared with before estradiol application; ##p < 0.01, compared with estradiol’s effect on TA-CA1 fEPSPs.

Gonadal hormones have been reported to modulate neural morphology and synaptic plasticity [43,44]. We wondered whether the differential levels of endogenous estrogen in male and female animals were involved in the difference in TA-CA1 LTP expression. We therefore compared TA-CA1 LTP evoked by TBS stimulation in slices from females in the proestrus phase (high estrogen) to the slices from the females in the diestrus phase (low estrogen) of the menstrual cycle. We found that the magnitude of TA-CA1 LTP was not significantly different between the two stages (Fig 4A). Furthermore, 17β-estradiol (1 nM) applied for 90 minutes prior to TBS did not alter the magnitude of TA-CA1 LTP recorded in slices from male rats (Fig 4B). Estradiol (1 nM) increased both TA-CA1 fEPSPs and SC-CA1 fEPSPs recorded in slices from male rats, but the effect was larger in the SC-CA1 pathway (Fig 4C).

**Fig 5:**
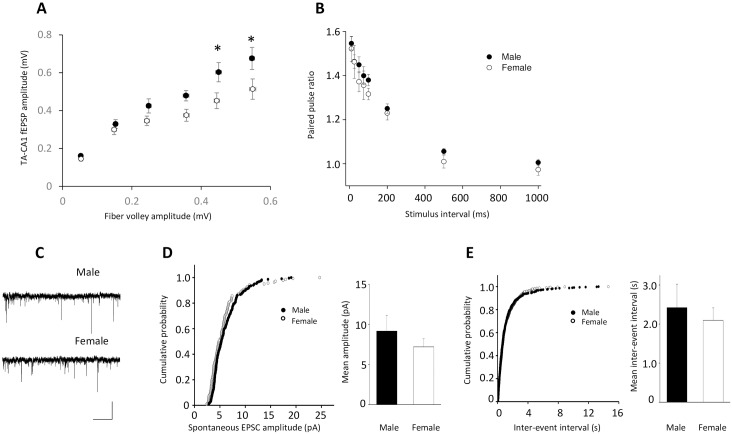
Comparison of basal synaptic transmission at TA-CA1 synapses in male and female rats. (A) A plot of TA-CA1 fEPSP slopes as a function of fiber volley amplitude revealed that slices from male animals exhibited a greater postsynaptic response at higher stimulus intensities than slices from female animals in response to similar presynaptic depolarization (male, n = 14 slices from 5 animals; female, n = 16 slices from 5 animals. *p<0.05). (B) Paired stimulation of TA-CA1 afferents at intervals of 10, 25, 50, 75, 100, 200, 500 and 1000 ms was given and paired pulse ratios (PPR) were calculated by dividing the peak amplitude of the second fEPSP by the peak amplitude of the first fEPSP. No differences in PPRs between males and females were seen (male, n = 15 slices from 5 animals; female, n = 11 slices from 5 animals). (C-E) Representative traces and data summaries show that neither the peak amplitude nor the frequency of spontaneous EPSCs recorded at the soma of CA1 pyramidal cells was different between males and females (mean amplitudes: male, 9.2 ± 1.6 pA, n = 6 cells from 1 animal; female, 7.2 ± 0.9 pA, n = 6 cells from 2 animals, t = 1.1, p > 0.05; mean inter-event intervals: male, 2.4 ± 0.6 s, n = 6 cells from 1 animal; female, 2.1 ± 0.2 s, n = 6 cells from 2 animals, t = 0.46, p > 0.05).

To investigate the mechanism underlying the sex difference in TA-CA1 LTP, we began by comparing basal excitatory synaptic transmission at TA–CA1 synapses between male and female animals. For this comparison, we first examined the efficiency of excitatory synaptic transmission at TA-CA1 synapses by plotting the TA-CA1 fEPSP peak amplitude as a function of the fiber volley. We found that, particularly at higher stimulus intensities, fEPSP amplitudes for a similar fiber volley were larger in males compared to females (Fig 5A). RMANOVA revealed significant overall effects for sex [F(1,168) = 18; p < 0.0001] and fiber volley amplitude [F(5,168) = 37; p < 0.0001], but no interaction [F(5,168) = 1.4; p > 0.05]. Bonferroni post-hoc tests indicated that EPSP amplitudes were significantly larger for males at the two highest fiber volley amplitudes. Presynaptic characteristics of TA–CA1 synapses were assessed by measuring the paired-pulse facilitation (PPF), which is the ratio of the peak amplitude of fEPSPs to two consecutive TA stimuli delivered at different time intervals. We observed that PPF was not significantly different at TA-CA1 synapses between male and female animals (Fig 5B), indicating that the probability of presynaptic release at these synapses was not affected by sex. We then tested whether spontaneous synaptic transmission in CA1 is different between male and female rats. Spontaneous EPSCs (sEPSCs) were recorded from the soma of CA1 pyramidal cells in whole-cell voltage clamp mode in the presence of picrotoxin (100 μM) and CGP52432 (4 μM) to block GABA_A_ and GABA_B_ receptors, respectively. Area CA3 and the dentate gyrus were cut from the slice to prevent spontaneous epileptiform discharges. We found that neither amplitude nor frequency of hippocampal CA1 sEPSCs was significantly different between male and female rats (Fig 5C–5E).

**Fig 6:**
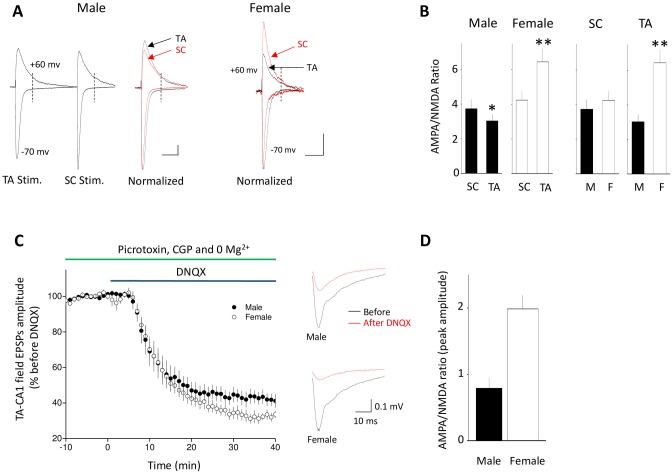
The ratio of AMPAR /NMDAR-mediated current at TA-CA1 synapses is different between male rats and female rats. (A) Representative and superimposed traces show inward and upward TA-CA1 and SC-CA1 EPSCs recorded at the same cell when cell membrane potential was held at either -70 mV or +60 mV to assess AMPA and NMDA currents, respectively. Left: example of the currents evoked by TA or SC stimulation; vertical dashed line indicates when the NMDA current was measured. Right: Normalized average responses from neurons in male and female rats. Scale bar: 30 pA, 100 ms. (B) Graphs of data summary in panel A. Left: Male rats had smaller AMPA/NMDA receptor ratios at TA versus SC synapses, while the opposite was seen in females. (male, 3.7±0.59 at SC-CA1 synapses, 3.0±0.43 at TA-CA1 synapses, n = 10 cells from 8 animals, t = 2.43; female, 4.2±0.57 at SC-CA1 synapses, 6.4±0.78 at TA-CA1 synapses, n = 12 cells from 8 animals, t = 3.11; paired *t*-tests).. Right: Comparisons of AMPA/NMDA receptor ratios showed that they were not different for SC-CA1 synapses, but that the ratio was significantly higher in females for TA-CA1 synapses (t = 0.61 for SC comparison; t = 3.61 for TA comparison; unpaired *t*-tests). * p < 0.05, ** p < 0.01 (C) Time course and representative traces show that DNQX largely blocked TA-CA1 fEPSPs in Mg^2+^-free ACSF containing picrotoxin and CGP52432 in slices from both male and female rats, revealing the NMDA component of the responses. (D) Graphs of summary data from panel C, comparing the ratio of DNQX-blocked non-NMDA component to the remaining NMDA component of TA-CA1 fEPSPs recorded 35–40 min after bath application DNQX in slices from male and female rats, respectively (the ratios were calculated from peak amplitude of the potentials: male, 0.81±0.21, n = 5 slices from 5 animals; female, 1.99±0.24, n = 6 slices from 5 animals.) The AMPA/NMDA ratio was significantly larger in females. ** t = 3.62, p < 0.01.

The number of postsynaptic NMDA receptors, or the ratio of functional AMPA to NMDA receptors, may influence the induction or expression of LTP. We therefore determined the ratio of AMPA receptor-mediated and NMDA receptor-mediated evoked EPSCs (AMPA/NMDA ratio) at TA-CA1 and SC-CA1 synapses in both male and female rats. We found that AMPA/NMDA ratio was smaller at TA-CA1 synapses than that at SC-CA1 synapses in male rats (Fig 6A and 6B), consistent with a previous report [45]. In contrast, the ratio was greater at TA-CA1 synapses than that at SC-CA1 synapses in female rats. Remarkably, female rats exhibited a much larger (213%) AMPA/NMDA ratio at TA-CA1 synapses compared to their male littermates, but there was no significant difference in the ratio at SC-CA1 synapses between males and females (Fig 6A and 6B). We next isolated the NMDA receptor-mediated component of TA-CA1 fEPSPs recorded extracellularly at CA1 SLM by blocking non-NMDA glutamate receptors with DNQX (50 μM) in magnesium-free saline and in the presence of picrotoxin (100 μM) and CGP52432 (4 μM). Consistent with the observations from whole-cell recordings, the AMPA /NMDA ratio at TA-CA1 fEPSPs was significantly smaller in male animals (Fig 6C and 6D).

**Fig 7:**
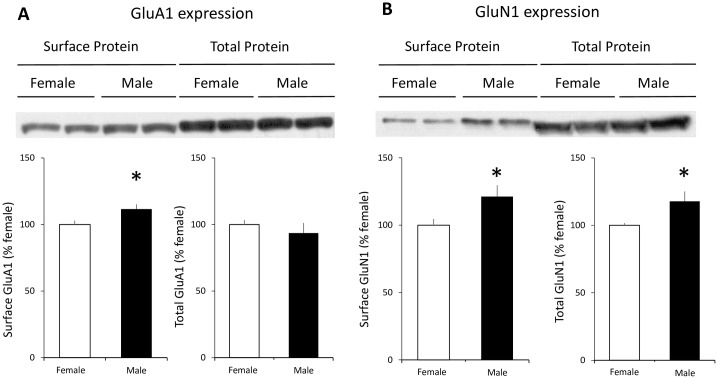
Male rats express more GluA1-containing AMPA receptors and NMDA receptors on the postsynaptic membrane surface of TA-CA1 synapses. (A) Upper: representative Western blots of SLM tissue wedges sub-dissected from acutely prepared hippocampal slices show that male animals expressed more surface, but not total, GluA1 at TA-CA1 synapses. Lower: data summary of 3 independent membrane surface protein biotinylation experiments from 6 pairs of littermate male and female rats (male surface GluA1: 111.6 ± 3.5% of female, n = 6, t = 2.34, p < 0.05; male total GluA1: 93.5 ± 7.7% of female, n = 6, t = 0.85, p > 0.05). (B) Upper: representative Western blots of SLM tissue wedges sub-dissected from acutely prepared hippocampal slices show that male animals expressed more surface and total GluN1 at TA-CA1 synapses. Lower: data summary of 3 independent membrane protein biotinylation experiments from 6 pairs of littermate male and female rats (male surface GluN1: 121.2 ± 8.4% of female, n = 6, t = 2.47, p < 0.05; male total GluN1: 117.8 ± 7.3% of female, n = 6, t = 2.39, p < 0.05). *p < 0.05.

A smaller AMPA/NMDA ratio could result from a smaller AMPA component or a larger NMDA component. To examine these possibilities, we assessed postsynaptic membrane surface, as well as total, expression of AMPA and NMDA receptors at TA-CA1 synapses in the hippocampal CA1 SLM region of male and female animals. The major forms of AMPA receptors in the hippocampus include GluA1 homomers as well as GluA1/2 and GluA2/3 heteromers [46,47]. The functional role of GluA1 and the phosphorylation of the subunit have been extensively investigated in AMPA receptor trafficking and LTP expression. We therefore examined surface GluA1 expression using synaptosomes isolated from CA1 SLM wedges. Surprisingly, we found that male rats expressed slightly, but significantly (12%), more surface GluA1 in the SLM region of CA1 than female animals (Fig 7A). Total GluA1 expression was not different in CA1 SLM between males and females. NMDARs are tetramers and are assembled from GluN1 and GluN2subunits. We compared the membrane surface and total GluN1 subunit expression in the CA1 SLM region between male and female animals. We found that male rats expressed markedly more surface (21%) and total GluN1 (18%) than female rats (Fig 7B). Taken together, the observation of more surface NMDA receptors and a relatively smaller surface receptor AMPA/NMDA ratio in male CA1 SLM provides a plausible explanation for our observation of greater TA-CA1 LTP recorded in the male rats.

## Discussion

Substantial research has been done describing the nature, and possible underlying mechanisms of, sex differences in cognition. Data from human studies reveal a consistent male advantage in a variety of spatial tasks [48,49]. However, the reports of sex effects on spatial ability in rodents are divergent and controversial. Sprague-Dawley rats, for example, show a substantially larger male advantage than any other strain [49]. In general, male rats outperform females in the radial arm maze and water maze tests, but in mice the male advantage in the radial arm maze is much less and females tend to outperform males in the water maze [50]. Our observations in the present study, using adolescent animals, are consistent with the majority of other studies showing that adult male Sprague-Dawley rats outperform adult females in the standard water maze test.

We observed that sex differences in hippocampal LTP depended upon both the pathway and the stimulation paradigm employed. We found that ELTP stimulation of the TA-CA1 pathway generated comparable LTP in males and females. In contrast, when LLTP stimulation paradigms were used, male rats exhibited larger magnitude HFS- and theta burst-induced TA-CA1 LTP than female rats. In our study, male and female rats expressed similar LTP at SC-CA1 synapses, suggesting that sex differences in LTP are synapse, as well as stimulation paradigm, specific. Overall, the differences we observed, while statistically significant, were not large. Recordings from littermates (Fig 3) provided the most convincing evidence for a sex difference in TA-CA1 LTP. In recordings made from nonlittermates (Fig 4) the sex difference was reduced, although TBS-LTP in the TA-CA1 pathway of females was still significantly smaller than for males when all the data were considered together. Other studies evaluating the effect of sex on SC-CA1 LTP have shown either no difference [31] or greater LTP in males[30,31], depending upon the stimulation paradigm that was employed. An additional complication in comparing studies is that, at least *in vivo*, it has been shown that the magnitude of SC-CA1 LTP varies across the estrous cycle [51]. Estrous status was not documented in either of the above-cited studies.

It has been postulated that gonadal hormone fluctuations across the female estrus cycle could account for inconsistent performance in spatial navigation tasks [52–54]. Substantial literature suggests that ovarian steroids, such as 17-β estradiol, modulate hippocampal dendritic spine density, synaptic activity and synaptic plasticity across the estrus cycle, with the highest level of excitatory effect during proestrus [55,56]. However, the behavioral impact of these changes is controversial. A study in humans showed superior spatial and nonspatial learning during the estrus (low estrogen) rather than proestrus phase (high estrogen) [54]. Another study observed that ovariectomized female rats outperformed intact female rats in radial maze and water maze tasks [57]. Interestingly, ‘sex-reversed’ female mice (which carry a Y chromosome) perform better in the MWM than normal females, suggesting an important role for male genotype (as opposed to hormonal complement) on hippocampus-dependent spatial learning [58]. Moreover, studies have demonstrated that sex differences are present in pre-pubertal children (as early as age 4 and in the same direction as in adults) in virtual reality-based tasks [59,60], indicating that sex differences in spatial learning and memory exist prior to puberty and do not appear to require the effects of sex hormones at puberty.

Electrophysiological studies have shown that estradiol can enhance baseline responses and LTP at SC-CA1 synapses in the hippocampus of either male or ovariectomized female rats [43,61]. To test whether endogenous estrogen plays a role on LTP expression at TA-CA1 synapses, we tested the effect of estradiol on SC-CA1 and TA-CA1 fEPSPs and LTP in slices from male rats, as well as comparing TA-CA1 LTP in females in proestrus and diestrus phases. We found that acute estradiol application increased baseline responses in the SC-CA1 connection, and slightly enhanced TA-CA1 responses. However, estradiol did not alter the amplitude of TA-CA1 LTP in males. We also observed that TA-CA1 LTP magnitude was not different between female rats in the two different stages of the menstrual cycle. These results are consistent with a recent study from Smith et al. that estradiol administration to ovariectomized female rats increased both AMPA and NMDA receptor-mediated currents, but did not increase LTP magnitude, at TA-CA1 synapses [62]. In sum, our results indicate that reduced LLTP expression at TA-CA1 synapses in female rats is likely not attributable to varying estrogen levels across the estrus cycle.

It is widely accepted that the medial temporal lobe is pivotal for encoding, consolidation and short-term storage of explicit memory. A recent elegant study using optogenetic and electrophysiological methods showed that hippocampal place cells receive direct input from grid cells, as well as from border cells and head-direction cells [63]. This observation is consistent with the idea that hippocampal place cells integrate both allocentric and egocentric information in guiding spatial navigation [64]. Grid cells have been found in all of the layers of the medial entorhinal cortex and are intermingled with other spatial cells in deep layers [63]. It is still not completely clear how the outputs of entorhinal grid cells are integrated in hippocampal CA regions to contribute to hippocampal place field and spatial navigation. Interestingly, Brun et al. showed that removing the tri-synaptic input (EC layer II-DG-CA3-CA1) did not affect CA1 place fields, suggesting that spatial information from the entorhinal cortex may bypass DG and CA3, and be conveyed directly to CA1 by the TA pathway originating in layer III of the entorhinal cortex. Further, behavioral studies have shown that the isolated TA-CA1 input is sufficient to support spatial recognition in the water maze, although both the tri-synaptic inputs and the TA-CA1 inputs are necessary for recall of remote locations or the trajectories towards these locations [34]. Thus, it is reasonable to hypothesize that sex differences in TA-CA1 plasticity could affect spatial memory.

NMDA receptors play a critical role in both LTP and in spatial learning and memory [65–67]. In CA1 pyramidal cells, NMDARs contain mainly GluN2A and/or GluN2B subunits in addition to GluN1. In NMDA receptor-dependent LTP, an NMDAR-mediated rise in postsynaptic Ca2+ activates a complex cascade of kinases and phosphatases that promotes AMPA receptor insertion in postsynaptic membranes and causes persistent changes in synaptic strength. We found that postsynaptic NMDA receptor expression was higher at TA-CA1 synapses in male rats, although male rats expressed slightly higher GluA1-containing AMPA receptors in SLM as well. Accordingly, the AMPA/NMDA receptor ratio was lower in males. Although the correlation between the AMPA/NMDA ratio and magnitude of LTP has not been systematically studied, the amount and duration of Ca^2+^ influx from NMDA receptor is critical to induce LTP or LTD [68]. In addition, an increase in AMPA receptor-mediated fast synaptic transmission and increase in the AMPA/NMDA receptor ratio occludes the induction of LTP [69] and impairs associative learning [67]. Thus, in males, greater surface expression of NMDA receptors may result in more Ca2+ entry, facilitating LTP induction. However, the role of AMPA/NMDA receptor ratios in inducing ELTP- versus LLTP-stimulated changes in synaptic strength remains to be further explored.

A key question is whether, and how, increased TA-CA1 LLTP magnitude translates into longer memory in the water maze task. Males and females showed similar memory at 24hrs after training, but males outperformed females at longer delays. Whether a sex difference in the biochemical mechanisms elicited by LLTP (but not ELTP) stimulation in the TA-CA1 connection is responsible for this difference is a subject for further research. To begin to answer this question, a future study will focus on making selective TA lesions to validate a role of this pathway in the sex difference we observed in memory consolidation. In addition, it will be important to demonstrate that the sex differences in TA-CA1 plasticity we observed in adolescent animals are also seen in adults.
